# Characterisation of European Field Goat Prion Isolates in Ovine PrP Overexpressing Transgenic Mice (Tgshp IX) Reveals Distinct Prion Strains

**DOI:** 10.3390/pathogens13080629

**Published:** 2024-07-27

**Authors:** Sonja Ernst, Romolo Nonno, Jan Langeveld, Olivier Andreoletti, Cristina Acin, Penelope Papasavva-Stylianou, Theodoros Sklaviadis, Pier Luigi Acutis, Lucien van Keulen, John Spiropoulos, Markus Keller, Martin H. Groschup, Christine Fast

**Affiliations:** 1 Friedrich-Loeffler-Institut, 17493 Greifswald-Isle of Riems, Germany; sonja.ernst@fli.de (S.E.);; 2 Department of Food Safety, Nutrition and Veterinary Public Health, Istituto Superiore di Sanità, 00161 Rome, Italy; 3 Wageningen BioVeterinary Research, Wageningen University & Research, P.O. Box 65, 8200 AB Lelystad, The Netherlands; 4 UMR INRAe/ENVT 1225, Interactions Hôtes Agents Pathogènes, Ecole Nationale Vétérinaire de Toulouse, 23 Chemin des Capelles, 31076 Toulouse, France; 5 Centro de Encefalopatías y Enfermedades Transmisibles Emergentes, Universidad de Zaragoza IA2 IIS Aragón, C/ Miguel Servet 177, 50013 Zaragoza, Spain; 6 Veterinary Services, Ministry of Agriculture, Rural Development and Environment, 1417 Nicosia, Cyprus; 7 School of Pharmacy, Aristotle University of Thessaloniki, University Campus, 54124 Thessaloniki, Greece; 8 Istituto Zooprofilattico Sperimentale del Piemonte, Liguria e Valle d’Aosta, Via Bologna 148, 10154 Torino, Italy; 9 Department of Pathology and Animal Science, APHA Weybridge, Addlestone KT15 3NB, Surrey, UK

**Keywords:** classical scrapie, atypical scrapie, CH1641-like, strain typing, goat, transgenic mice, PrP^Sc^, immunohistochemistry, Tgshp IX, BSE, bovine spongiform encephalopathy

## Abstract

After the detection of bovine spongiform encephalopathy (BSE), and a zoonotic transmissible spongiform encephalopathy (TSE) caused by the pathological prion protein (PrP^Sc^) in two goats, the investigation of goat prions became of greater interest. Therefore, a broad collection of European goat TSE isolates, including atypical scrapie, CH1641 and goat BSE as reference prion strains were biochemically characterised and subsequently inoculated into seven rodent models for further analysis (already published results of this comprehensive study are reviewed here for comparative reasons). We report here the histopathological and immunohistochemical data of this goat TSE panel, obtained after the first passage in Tgshp IX (tg-shARQ) mice, which overexpress the ovine prion protein. In addition to the clear-cut discrimination of all reference prion strains from the classical scrapie (CS) isolates, we were further able to determine three categories of CS strains. The investigation further indicates the occurrence of sub-strains that slightly resemble distant TSE strains, such as BSE or CH1641, reinforcing the theory that CS is not a single strain but a mixture of sub-strains, existing at varying extents in one isolate. This study further proved that Tgshp IX is a potent and reliable tool for the in-depth characterisation of prion strains.

## 1. Introduction

The group of transmissible spongiform encephalopathies (TSEs) encompasses several infectious, spontaneous or hereditary diseases affecting both humans and animals [1]. TSEs have been reported in livestock and wild animal populations, i.e., cattle (bovine spongiform encephalopathy, BSE), small ruminants (scrapie), cervids (Chronic Wasting Disease, CWD) and most recently camelids (camel prion disease, CPD) [2,3,4,5]. All TSEs are caused by the accumulation of an infectious, misfolded, partially protease-resistant and insoluble isoform (PrP^Sc^) of the otherwise cellular prion protein (PrP^C^) [6].

In contrast to all other TSEs, BSE prions easily overcome the species barrier, inducing disease in sheep (ovBSE), goat (gtBSE) and even humans (variant Creutzfeldt–Jakob disease, vCJD) [7,8,9]. While no natural cases of ovBSE have been detected, two reports of naturally infected goats have been published so far [9]. Therefore, the possibility that BSE circulates in sheep and goat populations cannot be fully excluded.

BSE, scrapie and possibly also CWD not only occur in their classical infectious forms but also as atypical, sporadic versions. Atypical scrapie (aka Nor98), H- and L-type atypical BSE and presumably red deer and moose CWD of northern European cervids are viewed as spontaneous age-dependent and most probably non-contagious diseases [10,11,12,13,14,15]. Furthermore, intensive research to better characterise the nature of prion infectivity revealed the occurrence of so-called “prion strains”, whose properties are encoded on the specific conformation of PrP^Sc^ [16]. It is hypothesised that conformational changes at biologically active sites are a necessity to overcome the transmission barrier of an otherwise cloned replicate of PrP^Sc^ (“deformed templating hypothesis”). The conformational changes can subsequently result in biochemical and histopathological differences and define a new strain [16,17]. Another hypothesis (“conformational selection model”) suggests that strains in general consist of a collection of different molecular PrP^Sc^ species, whereof the dominant one determines the pathological and biochemical features. When a strain binds to a yet unknown substrate or host, a less dominant molecule might be selected and preferably propagated, resulting in new patterns and subsequently determining a new strain [17,18].

Historically, wild-type mice were used for strain typing, and the results relied on the analysis of incubation periods and lesion profiles by using a combination of wild-type mice [7,19]. More recently transgenic mice were introduced for strain typing purposes, and the propagated prion strain was characterised by additional biochemical and immunohistochemical examinations. In the last years, in particular, bank vole and diverse transgenic mouse models overexpressing PrP^C^ of different species were established [20,21,22,23]. Western blot (WB) analyses of proteinase K-digested PrP^Sc^ and immunohistochemistry (IHC) not only allow for the discrimination of BSE and scrapie but also the differentiation of classical and atypical TSE forms according to their specific PrP^Sc^ accumulation (PrP^Sc^ profile) and WB PrP^Sc^ banding patterns. So far, the definition of a new strain depends on its phenotypic characteristics of the field cases (clinical signs, lesion/PrP^Sc^ profile, biochemical and molecular characteristics) and/or its biological properties obtained after in vivo studies in experimental rodent models (attack rate, incubation period, lesion/PrP^Sc^ profile and WB banding pattern).

In European classical scrapie, at least five phenotypically different prion strains have been identified so far, but the exact number still remains unknown [16,20,22,24]. Additionally, the diversity of goat scrapie strains in Europe has lately been investigated using bioassays on different rodent models, leading to the characterisation of at least four goat classical scrapie types, in addition to atypical scrapie [20,22,25]. Prions are highly variable and adaptive: besides BSE, several classical scrapie strains also have been reported to cross species barriers [26,27,28]; hence, an intrinsic potential of PrP^Sc^ to become zoonotic has to be considered. Thus, it is of utmost importance to identify and characterise circulating prion strains in the field not only to improve risk assessment but also to obtain clear-cut differences between classical scrapie and BSE strains to easily recognise any classical BSE prions that may circulate in the field. Recently, ovine PrP^C^ overexpressing transgenic mice (Tgshp IX) have shown to be a reliable tool to discriminate different scrapie prions as well as ovBSE and BSE [23]. In addition, transmission efficiency and biochemical data of this mouse model were successfully used in a comprehensive study addressing the European goat TSE strains circulating in the field, analysed by Nonno and colleagues [20]. The additional examination of the lesion and PrP^Sc^ profiles induced by these isolates in Tgshp IX (tg-shARQ) mice presented here expands the knowledge of the underlying prion strains.

## 2. Materials and Methods

The here-used natural goat TSE isolates were previously described [20,22,25]. The samples were collected to identify the geographical, pathological and genetic variability of naturally circulating caprine PrP^Sc^. Table 1 gives an overview of all isolates used and additional information on the country of origin, genotype and PrP^Sc^ type. Briefly, the sample set used for inoculation consists of, in total, 35 isolates, including 29 brain tissues from field TSE cases in goats, one brain sample from an experimentally BSE-infected goat, and two brain inoculates of goats experimentally infected with scrapie as well as three lymph node isolates. The field samples were derived from the active TSE-EU surveillance programme and were detected as positive by approved screening tests with subsequent BSE–scrapie discrimination. In order to gain comparable results, from each goat brain, one 50% macerate in water was produced in one laboratory under sterile conditions as previously reported [20]. These brain homogenates were stored at −80 °C and aliquots were sent to all partner laboratories. The detailed analysis of the WB banding pattern of the field cases was recently reported [25] as well as the results of the different rodent models involved in this comprehensive study [20,22].

The competent authority of the Federal State of Mecklenburg-Western Pomerania, Germany approved the here-described animal experiments (reference number LALLF 7221.3-2.1-012/03 and 7221.3-2.1-027/02) based on national and European legislation, i.e., Directive 2010/63/EU of the European Parliament and of the council on the protection of animals used for scientific purposes. For this study’s bioassay, a transgenic mouse model (Tgshp IX; tg-shARQ) devoid of murine PrP was used. The mice were generated at the Friedrich-Loeffler-Institut (FLI), overexpressing the ARQ allele of ovine PrP two- to four-fold, on a B6CBAx129Ola genetic background. In advance of the inoculation, the 50% brain macerates were adjusted to 10% homogenates in 0.9% sodium chloride. Per isolate, 15 six- to eight-week-old mice were inoculated intracerebrally with 20–30 µL of the brain tissue homogenates. Animals were clinically examined and scored at least twice a week until the clinical onset of the disease. Before the disease became too heavy and compromised the animal’s welfare, mice were euthanised. Animals not showing any signs of disease onset were euthanised at the defined endpoint of the experiment at 730 dpi. At necropsy, brains from inoculated mice were removed and divided longitudinally in the paramedian. The smaller half of the brain was stored at −20 °C and used for the WB analysis published before [20]. The second half of the brain was fixated in 4% buffered formalin (FFPE). The brains of the animals found dead were stored at −20 °C only. FFPE samples were prepared for histopathological investigation according to a standardised protocol [19,23]. Brains were cut at four different coronal levels to reveal the cerebellum with the brain stem, midbrain nuclei, thalamus, hypothalamus, hippocampus, corpus callosum, frontal cortex and septum (Figure S1). The segments were paraffin-embedded and further processed in 3 µm sections on Superfrost plus slides (Menzel, Darmstadt, Germany).

Only animals that scored positive or inconclusive after WB analysis were used for lesion profiling and the PrP^Sc^ profile via IHC. The incubation period is defined as the time frame between inoculation of the animal and the time point of death (days) post infection. All positive animals were used for the calculation of the mean and standard deviations. The attack rate was calculated using the incubation period of the first positive mouse as the minimum time for transmission of the disease. All mice with a shorter period of time after infection were not considered.

Spongiform neuropathology was evaluated after haematoxylin and eosin staining and according to a standardised procedure [19,23], including the analysis of well-defined regions of grey- and white-matter areas from the medulla oblongata, cerebellum, mesencephalon, diencephalon and telencephalon. Table 2 contains a detailed list of all investigated brain regions. A score from weak to severe (scored 1–3) was used to describe the lesion profile.

Immunodetection via IHC was performed as recently published [30], adding laboratory-specific modifications. After rehydration in graded alcohol, the slides were incubated in 98% formic acid for 15 min and subsequently rinsed with tap water for 5 min. To inhibit endogen peroxidase, 3% H_2_O_2_ in methanol was applied before autoclaving the slides for 20 min at 121 °C in citrate buffer (pH 6.1). A 10 min serum block using 50% goat serum preceded the application of the primary antibody R145 (APHA scientific, Addlestone, UK), a rat anti-PrP-specific monoclonal antibody diluted at 1:750 in 10% goat serum. Afterwards, a secondary biotinylated anti-rat antibody (Vector Laboratories Inc., Newark, CA, USA) was applied for 30 min, followed by an avidin–biotin complex (ABC, vectastain/Vector Laboratories Inc., Newark, CA, USA) for another 30 min. To stain and counterstain the antibody-bound PrP chromogen, diaminobenzidine tetrahydrochloride (Vector Laboratories Inc., Newark, CA, USA) and Mayer’s haematoxylin were used. The slides were finally dehydrated in graded alcohol and cover-slipped for subsequent investigation by light microscopy. The same regions for the lesion profiling were evaluated (Table 2). Additional regions with remarkable reaction patterns were documented as well. A score from weak to severe (score 1–3) was used to describe the PrP^Sc^ profile.

## 3. Results

Within the context of the so-called GoatBSE (FOOD-CT-2006-36353) project, in total, 35 goat TSE isolates from seven European countries, including the UK, were inoculated for a comparative analysis in several transgenic mouse models and bank voles. For all bioassays involved, the same homogenate was used, which was prepared in one laboratory and distributed in aliquots to the participating partners. The biochemical phenotypes of these homogenates are described by Langeveld and colleagues [25], and the biomolecular analysis of the brains of the rodent models after the first passage are summarised in Nonno et al. [20]. Additionally, a detailed examination of the tg-bov (Tg110) and tg-gtARQ (Tg501) bioassays was recently published [22]. Here, we report the data obtained by the histopathological and immunohistochemical investigation of the also involved Tgshp IX (tg-shARQ) model. To put the data from our study into context, the data of the investigation of the field isolates, transmission features and biochemical analysis of all rodent models as well as the in-depth investigation of tg-gtARQ (tg501) and tg-bov (tg110) mice were reviewed. Table 3 gives a full overview of all studies, thus enabling a direct comparison of the outcome of these comprehensive animal experiments [20,22,25].

### 3.1. Transmission Profile

Transmission of all isolates to Tgshp IX mice was successful after the first passage. Taking both attack rate and incubation period (IP) into consideration, most of the classical scrapie (CS) isolates can be categorised into groups, showing either “short”, “intermediate” or “long” IPs as shown in Table 1 and Table S1. The majority of the CS isolates had IPs of less than 300 days post inoculation (dpi), combined with mostly high to complete attack rates, resulting in an average attack rate of 90.03%. Isolates with an intermediate IP ranging from 321 dpi to 399 dpi were mostly associated with incomplete attack rates (mean 82.65%). Eventually, isolates with long incubation periods of up to 503 dpi (isolate UKD2) also showed low attack rates (mean 72.95%).

Only a few isolates showed slight deviations from this scheme. Isolates F16 (55.56%) and UKA1 (66.67%) had exceptionally low attack rates, but still short IPs of 270 dpi and 281 dpi, respectively, and were therefore grouped in Category A. Isolates I3 (464 dpi), I7 (417 dpi) and UKB1 (418 dpi) showed complete attack rates, but long IPs, and were therefore grouped in Category C. In Figure S2 the correlation between attack rate, incubation period and scrapie strain categories including the reference controls is shown.

### 3.2. Lesion Profile

As proven by standardised strain typing studies in conventional rodent models, the histopathologic examination of nine grey matter and three white matter areas (Table 2, Supplementary Figure S1) of the central nervous system creates a lesion profile in experimental mouse transmission studies that can be used for further characterisation [19]. In addition to the aforementioned areas of white and grey matter, the corpus callosum (CC) was examined here.

Vacuolar lesions were induced by all examined isolates, to different extents and were scored from 1 as mild to 3 as severe. Plaque formations were induced by 22 isolates in different neuroanatomic areas, with the highest frequencies in the CC. This was a characteristic pattern with BSE, but also detectable in CS isolates F10 and UKA2. Individual mice also showed multifocally in different brain regions signs of mild up to moderate inflammatory processes. Considering all results, the encephalitis and the lesion profile as well as the quality and quantity of the plaque formations, no specific strain-related information was obtained that allowed for the discrimination of isolates from one another. However, areas with severe lesions always indicated a severe PrP^Sc^ accumulation (Figure S3), and plaques seen in H&E staining were in most cases also detectable in IHC.

### 3.3. PrP^Sc^ Profile

The same neuroanatomic areas as examined for the lesion profile were taken into consideration to create the PrP^Sc^ profile that recognises the amount of PrP^Sc^ per area from 1 as mild to 3 as severe. However, for TSE discrimination purposes, further regions were targeted, such as the molecular and granular layer of the cerebellum and the CC (Table 2).

#### 3.3.1. PrP^Sc^ Profile of Atypical Scrapie, Caprine BSE and CH1641 (Reference Strains)

**Figure 1:**
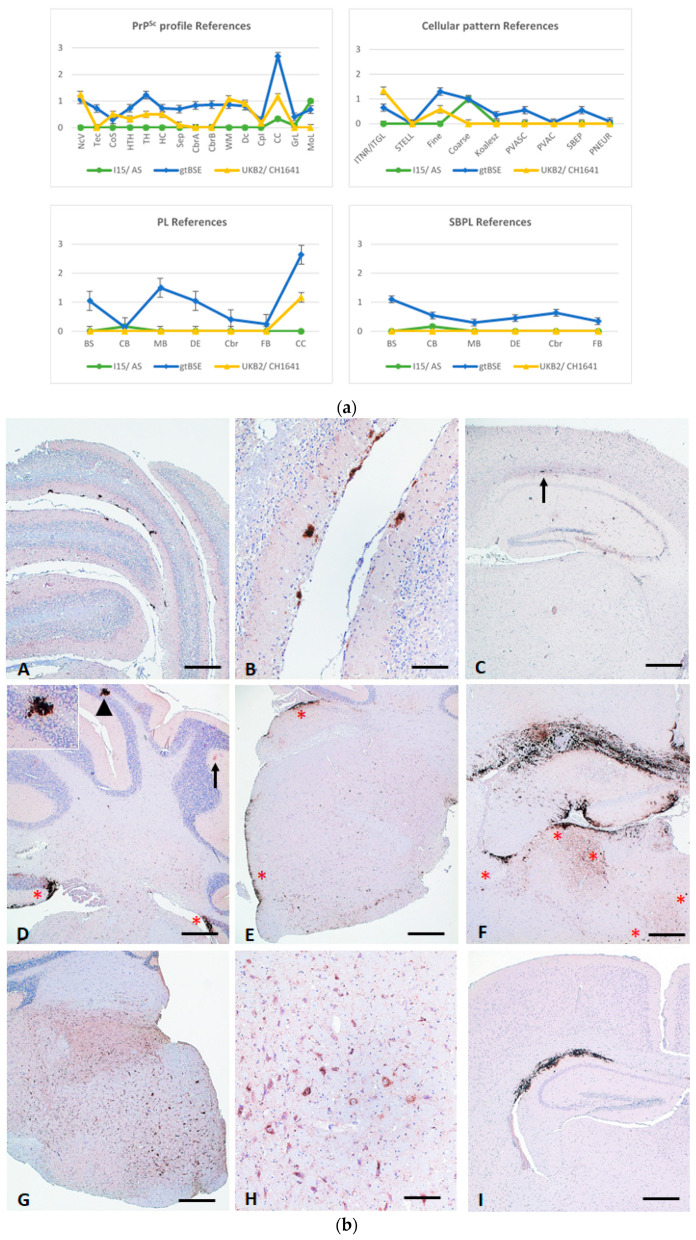
(**a**). PrP^Sc^ profile and cellular PrP^Sc^ deposition pattern of reference prion strains. The Tgshp IX PrP^Sc^ profile and deposition pattern of the three reference isolates atypical scrapie (AS), caprine BSE (gtBSE) and CH1641 used in the study are presented here. The Tgshp IX model allows for a clear-cut discrimination of all isolates in particular by including the granular and molecular layers of the cerebellum and corpus callosum in the PrP^Sc^ profile as well as the overall cellular reaction pattern. NcV = vestibular nuclei of medulla; Tec = tectum of cerebellum; CoS = cortex of the superior colliculus; HTH = hypothalamus; TH = thalamus; HC = hippocampus; Sep = septal nuclei; CbrA = cerebral cortex (at the level of thalamus and hypothalamus); CbrB = cerebral cortex (at the level of the septal nuclei); WM = cerebellar white matter; Dc = white matter in decussation fibres; DpI = internal capsule; CC = corpus callosum; GrL = granular layer of the cerebellar cortex; MoL = molecular layer of the cerebellar cortex; BS = brain stem; CB = cerebellum; MB = midbrain; DE = diencephalon; Cbr = cerebrum; FB = forebrain, CC = corpus callosum. Error bars indicate the standard error of means. (**b**). Distinct differences between the reference prion strains are seen in cerebellum and diencephalon of representative tg-shARQ (Tgshp IX) mice per group: (**A**–**C**) atypical scrapie with distinct PrP^Sc^ accumulation only in molecular layer of cerebellum and spurious amounts in corpus callosum (arrow); (**D**–**F**) goat BSE with PrP^Sc^ accumulation in granular (arrowhead and inlet) and molecular layer (arrow) of cerebellum as well as distinct subpial reaction pattern in brain stem and cerebellum (**E**, stars), as well as massive PrP^Sc^ depositions in corpus callosum and thalamus, including widespread plaque/plaque-like formations and subpial reaction pattern (**F**, stars); (**G**–**I**) CH1641 with a distinct intraneuronal reaction pattern in brain stem associated with fine extracellular PrP^Sc^ deposition, and in addition, moderate PrP^Sc^ accumulation in corpus callosum. PrP immunohistochemistry mab R145, Bar A, C, D, E, G and I 100 µm, B, H 50 µm.

The PrP^Sc^ profiles of all isolates allowed for a clear-cut discrimination of atypical scrapie (AS) and caprine BSE (gtBSE) from all other CS isolates and gave a clear indication for CH1641 (Figure 1a,b).

PrP^Sc^ deposition in AS was almost strictly confined to the molecular layer of the cerebellum with only very mild accumulation in the corpus callosum (CC), while all other brain areas remained negative.

Characteristically for caprine BSE is the wide distribution of PrP^Sc^ deposition in all examined areas to different extents. The most severe accumulation was seen in the brain stem region, the thalamic nuclei and especially the CC. Further, the prominent, widespread and significant plaque and plaque-like formations, as well as severe subpial PrP^Sc^ depositions, made BSE clearly distinguishable. In addition, gtBSE was the only isolate that induced PrP^Sc^ accumulation in both the granular and the molecular layer of the cerebellum. It is noteworthy at this point that this pattern is very similar to the data reported for BSE-and ovine BSE infected Tgshp IX mice [23], also showing a widespread distribution with prominent PL/PL-like patterns, in particular in CC and in a subpial reaction pattern. In addition, the characteristic PrP^Sc^ deposition in the granular layer of the cerebellum is also seen.

Even though the PrP^Sc^ profile of the CH1641 (UKB2) isolate is distinct, discrimination for this scrapie type was not as clear-cut as for AS and BSE. However, the prominently seen and widely distributed intracellular (ITNR) PrP^Sc^ accumulation with only a fine extracellular deposition pattern was singular.

#### 3.3.2. Geographical Analysis along PrP^Sc^ Profile of CS Isolates

At first, a geographical analysis was performed, comparing the PrP^Sc^ profiles of isolates from the same country of origin. Isolates from Italy, Greece, Spain, Cyprus, the Netherlands and the UK revealed matching profiles of all isolates when compared to those of the same country (Figure S4). Differences seen are all attributed to the total amount of PrP^Sc^, but not to the profile. However, the comparison of the French isolates revealed a singular profile for isolate F16, while all others showed close homology.

**Figure 2:**
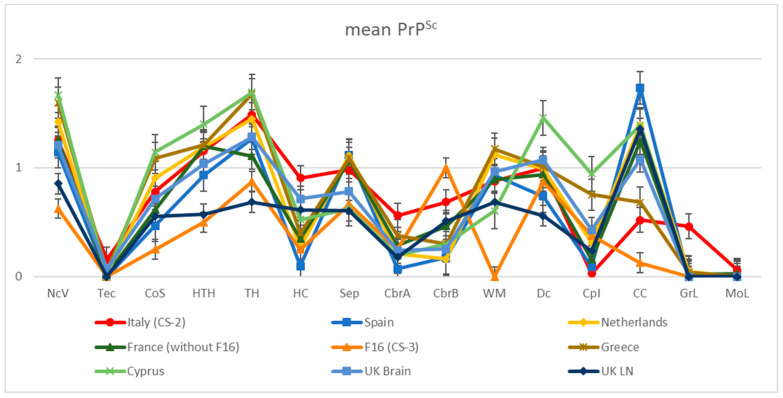
Mean PrP^Sc^ profile summarising the isolates per country with the exception of isolate F16 which is presented separately. Comparative analysis of the mean PrP^Sc^ profile of each country. Means from Spain, the Netherlands, France, Greece and Cyprus show a wide homology with peaks at the same brain regions. However, the Italian mean is clearly distinguishable due to its low PrP^Sc^ accumulation and the heightened peak at the level of the granular layer of the cerebellum. F16 showed an unique PrP^Sc^ profile with an almost complete lack of PrP^Sc^ accumulation in CC and WM and a distinct peak in CbrB. NcV = vestibular nuclei of medulla; Tec = tectum of cerebellum; CoS = cortex of the superior colliculus; HTH = hypothalamus; TH = thalamus; HC = hippocampus; Sep = septal nuclei; CbrA = cerebral cortex (at the level of thalamus and hypothalamus); CbrB = cerebral cortex (at the level of the septal nuclei); WM = cerebellar white matter; Dc = white matter in decussation fibres; DpI = internal capsule; CC = corpus callosum; GrL = granular layer of the cerebellar cortex; MoL = molecular layer of the cerebellar cortex. Error bars indicate the standard error of means.

Subsequently, for better comparison one mean PrP^Sc^ profile per country was calculated, whereas the French mean excludes the singular isolate F16. In doing so, it was obvious that the PrP^Sc^ profiles of the Greece, Spain, Cyprian, Dutch, the UK and the remaining French means were almost identical and will be summarised as the EU mean below. However, besides isolate F16, the Italian samples also revealed a unique profile (Figure 2).

**Figure 3:**
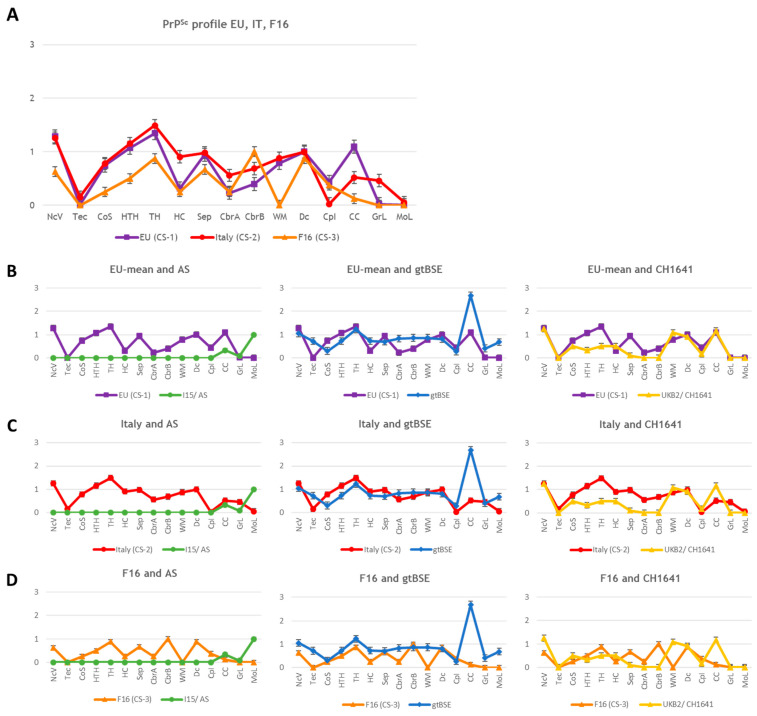
PrP^Sc^ profiles of EU mean (CS-1), Italian isolates (CS-2) and F16 (CS-3) compared to reference strains. Country means of Spain, the Netherlands, France, Greece, Cyprus and the UK were summarised to one European mean (EU mean) building category CS-1. The Italian isolates form the category CS-2, and the single F16 isolate is categorised as CS-3. (**A**) Comparative analysis of the three categories reveals distinct profiles, especially at the level of the corpus callosum (CC) and the granular layer of the cerebellum (GrL). (**B**–**D**) All categories were compared to reference strains atypical scrapie (AS), caprine BSE (gtBSE) and CH1641. (**B**) CS-1 was clearly distinguishable from AS and gtBSE, while CH1641 showed more overlapping features to CS-1 in most brain areas investigated, but the lack of PrP^Sc^ in the cortex and septum is striking. (**C**) A clear-cut discrimination from all three reference sequences was possible for CS-2. (**D**) CS-3 was clearly distinguishable from all references. NcV = vestibular nuclei of medulla; Tec = tectum of cerebellum; CoS = cortex of the superior colliculus; HTH = hypothalamus; TH = thalamus; HC = hippocampus; Sep = septal nuclei; CbrA = cerebral cortex (at the level of thalamus and hypothalamus); CbrB = cerebral cortex (at the level of the septal nuclei); WM = cerebellar white matter; Dc = white matter in decussation fibres; DpI = internal capsule; CC = corpus callosum; GrL = granular layer of the cerebellar cortex; MoL = molecular layer of the cerebellar cortex. Error bars indicate the standard error of means.

**Figure 4:**
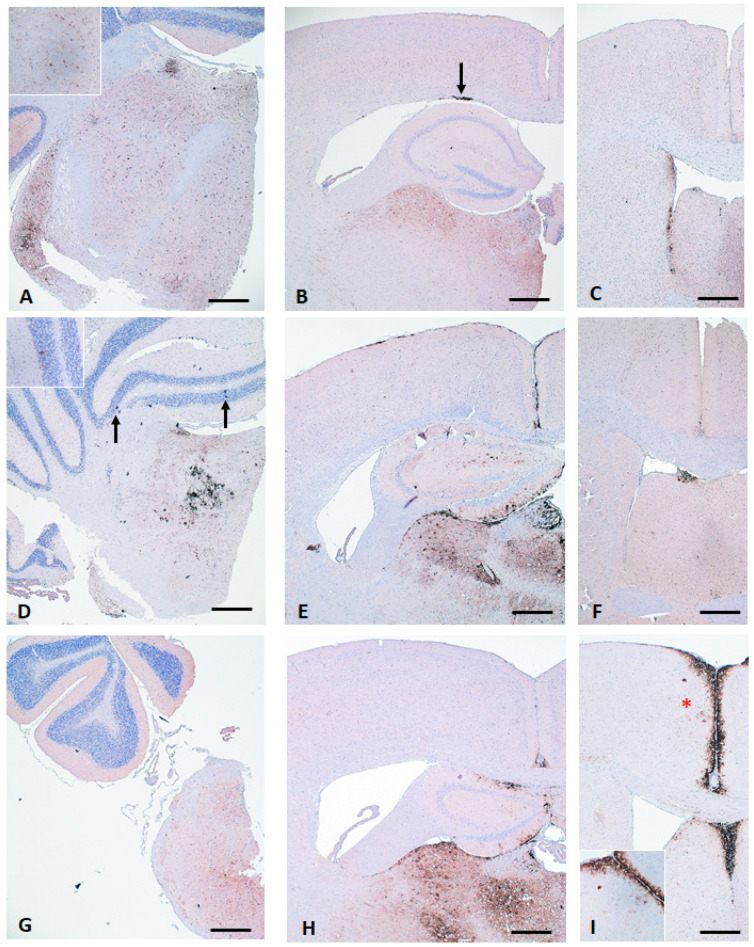
Distinct differences between classical scrapie strains (EU mean, Italy and F16) are visible in cerebellum/brain stem, diencephalon and cerebrum (at level of septum, CbrB) of representative tg-shARQ (Tgshp IX) mice per group: (**A**–**C**) EU mean (CS-1) showing a variable PrP^Sc^ deposition pattern, including a clear involvement of corpus callosum, and single areas even show similarities to CH1641 (i.e., distinct intraneuronal reaction pattern in brain stem; see inlet), but CbrB remain free of PrP^Sc^; (**D**–**F**) Italian cases (CS-2) with distinct PrP^Sc^ accumulation in granular layer of cerebellum (arrows, inlet), variable reaction pattern in diencephalon, but PrP^Sc^ is neither to be found in corpus callosum nor CbrB; (**G**–**I**) F16 (CS-3), no PrP^Sc^ accumulation in cerebellum and corpus callosum, but distinct deposition in CbrB (stars, inlet); immunohistochemistry mab R145, Bar 100 µm, inlets 50 µm.

The so-called EU mean can be characterised by distinct PrP^Sc^ accumulation mostly in the vestibular nuclei of the medulla and the hypothalamic and thalamic nuclei as well as in the CC. Slightly lower PrP^Sc^ depositions were seen in the septum (Sep), the midbrain and the white matter of the cerebellum, while the cortex remained mostly negative. In the molecular and granular layer of the cerebellum, no PrP^Sc^ was seen. The mean Italian PrP^Sc^ profile showed in most regions a similar profile to the EU mean. However, characteristic of these isolates is the peak in the granular layer of the cerebellum (GrL) and only weak accumulation in the CC. The F16 deviated from all other isolates, showing no deposits in the white matter, or the granular or molecular layer of the cerebellum, and only sporadic PrP^Sc^ accumulation in the CC. However, a distinctive peak was seen in the cerebral cortex at the level of the septal nuclei (CbrB). In total, three distinct CS profiles (CS-1–3) were distinguishable in the Tgshp IX (tg-shARQ) mouse model and can be clearly discriminated from the reference prion strains (Figure 3A–D and Figure 4).

In addition to the investigated brain samples, three lymph node isolates were also analysed (UKA1, UKB1 and UKC1). The PrP^Sc^ profiles of UKB1 and UKC1 showed close homology, and when compared to the EU mean, they revealed close similarities with only milder PrP^Sc^ accumulation in the thalamic and hypothalamic nuclei. Interestingly, UKA1 shows a unique PrP^Sc^ profile, deviating vastly from all other CS isolates as well as AS, BSE and CH1641-like isolates. Besides low peaks in the CbrB and the CC, all investigated regions remained negative.

### 3.4. Cellular Patterns and Plaque and Plaque-like Formations in CS Isolates

Several cellular patterns were detected at different frequencies of occurrence. Almost all isolates revealed prominent ITNR depositions confined to the caudal brain areas and predominantly seen in the brain stem and white matter of the cerebellum and only occasionally found in the midbrain and thalamus. Fine extracellular PrP^Sc^ depositions could be found in all investigated brain areas. Stellate, subependymal and perineuronal deposits were seen incoherently and mostly in severely positive animals that in general showed a variable mixture of cellular reaction patterns.

More indicative was the analysis of extracellular coarse, coalescing, perivascular and perivacuolar PrP^Sc^ deposits (Figure S5A,B).

- Coarse deposits were widely distributed and most severe in all Cyprian isolates as well as G2. However, in French isolates, including F16, such a pattern was rarely seen. Accordingly, the coarse deposits of the Cyprian isolates often transitioned into a coalescing pattern, which is only rarely seen in most of the other isolates and never in French isolates. In general, the coalescing pattern is mostly confined to the CC.
- Perivascular deposits are often associated with severely affected areas and are predominantly seen in the Cyprian and most Italian isolates.
- Perivacuolar deposits were found only occasionally and often parallel to perivascular patterns in areas with severe fine to coarse accumulation. Perivacuolar PrP^Sc^ were most prominent in isolate G2.

In general, plaque and plaque-like formations (PL) were seen at lesser extents in all CS isolates compared to the gtBSE isolate (Figure S5B). For comparative analysis, the midbrain, diencephalon, forebrain and cortex were combined into one region, called the “rostral brain”, excluding the CC, which is analysed as a separate region. Further regions are the brain stem and the cerebellum. The most important results are as follows:

- The rostral brain region only showed mild PL depositions in all isolates investigated.
- Mild numbers of PL depositions were seen in the brain stem, with the exception of the Italian samples, which in turn showed only minor amounts of PL depositions in the CC. In particular, isolates I3, I5 and I11 were most affected by PL deposits in the brain stem with a relatively lesser extent in the rostral brain.
- The CC is the area where most PL depositions were detectable, at mostly intermediate to severe extents induced by most of the CS isolates as well as gtBSE. In contrast, the Italian isolates, F16, G1 and G4, showed no or only mild PL deposits in this area.
- The cerebellum of most isolates was negative, and only a few showed mild PL formations.

In most groups, the PrP^Sc^ profile and the cellular reaction pattern were in good agreement among the mice per group, and deviations were only seen in the quantity of the detectable amount of PrP^Sc^ in single animals. An exception was isolate F11 which induced two distinct patterns in the group of inoculated mice. In three of the total nine positive animals, prominent and multifocal PL formations were seen across all brain areas, which slightly resembled a BSE-like pattern. Besides the prominent PL formations, these mice also showed distinct PrP^Sc^ accumulation in the hypothalamic nuclei and in the CC. On the contrary, the cellular pattern of the remaining mice could be described as CH1641-like, showing heavy intracellular PrP^Sc^ deposition in caudal brain areas, including the white matter of the cerebellum, the brain stem and the midbrain.

Taken together, using the Tgshp IX mouse model we were able to discriminate three CS groups: EU mean (CS-1), Italy (CS-2) and F16 (CS-3). All can be clearly discriminated from all reference strains. For example,

- None of the CS isolates induced a PrP^Sc^ accumulation in the molecular layer of the cerebellum typical for AS.
- The gtBSE typical simultaneous PrP^Sc^ deposition in the granular and molecular layer of the cerebellum was not induced by any of the CS isolates. In addition, the characteristic widespread SBPL and PL-like reaction pattern induced by gtBSE was also not achieved by any of the CS isolates.
- Although some of the CS isolates (S2, S3, F11, UKA1) and to a lesser extent isolates N3, F3 and UKD2 have single brain regions with a distinctive ITNR reaction pattern associated with a fine extracellular PrP^Sc^ accumulation, this pattern is unique in its extent only for CH1641.

An overview of the most important discriminatory parameters of all three CS categories as well as references strains AS, gtBSE and CH1641 is given in Table 4.

## 4. Discussion

It is widely accepted that classical scrapie in sheep and goats can be induced by several prion strains, whereas the exact number not only remains an enigma [20,24], but there are also indications that most field isolates contain sub-strains, which could influence the transmissibility of a classical scrapie isolate [20,22]. Besides analysing field isolates, the passage of the isolates in question in different rodent models has proven a crucial tool for an in-depth characterisation of TSE isolates [19,23]. Hence, to characterise circulating goat scrapie strains, a vast collection of goat field scrapie isolates from all over Europe, which includes atypical scrapie as well as experimental caprine BSE (gtBSE) and CH1641, were inoculated in a panel of conventional (RIII) and transgenic mice overexpressing PrP^C^ of different species (Tg110, Tg338, Tga20, Tgshp IX and Tg501) as well as bank voles. To further improve this comprehensive and comparative approach, all participating laboratories worked with the same inocula, which were prepared in one laboratory and distributed in aliquots to all partners. In addition, these homogenates were thoroughly characterised by biochemical examinations, indicating the presence of at least two prion strains [25]. Establishing the “transmission efficiency” as an additional parameter for characterising the ability of prion strains to overcome a transmission barrier, Nonno and colleagues were able to categorise these field isolates into up to four different categories, whereas two of the categories were discussed as subgroups of the second category [20]. They further proposed the combination of three different mouse models Tgshp IX (tg-shARQ)/tg-gtARQ (Tg501), tg-bov (Tg110) and tg-shVRQ (Tg338) as these delivered the most reliable results for scrapie strain typing and TSE discrimination [20]. Recently, Marin-Moreno and colleagues confirmed these results through a thorough analysis of the tg-gtARQ and tg-bov mouse model [22]. To round up this strain-related information, we present here the detailed strain typing data from the Tgshp IX mouse model based on lesion and PrP^Sc^ profiles of all 35 goat isolates, including atypical scrapie, caprine BSE and CH1641, which were considered reference control strains. In principle, our data support the categorisation of these field isolates, thus predicting the presence of several CS strains circulating in European goat populations. However, based on the Tgshp IX mouse model, we propose to classify the CS isolates into three categories, in which CS-1 contains the most isolates which were found all over Europe, except in Italy. The Italian isolates could all be summed up in category CS-2 whereas CS-3 consists of one single isolate from France (F16).

An initial investigation of attack rates and mean incubation periods in Tgshp IX mice gave the first indications of a prion strain variation associated with the different field isolates. Most isolates, later classified as CS-1, predominantly had high to complete attack rates and short incubation periods. In contrast, the Italian isolates summarised in CS-2 showed reduced attack rates and longer incubation periods. Moreover, the F16 isolate (CS-3) completely fell out of this classification scheme with a reduced attack rate and short incubation periods, which is also made clear by the presentation of the correlation between attack rate and incubation period. However, it has to be kept in mind that these parameters need to be interpreted with caution, as several selective factors directly impact prion strain transmission. For example, it is known that the dose of exposure of the infectious agent influences disease progression. However, the infectious titre, and thus the amount of PrP^Sc^ in field isolates, often remains unknown and varies among brain areas. Therefore, isolates with low infectious titres could result in poor transmission compared to isolates with high PrP^Sc^ amounts, assuming the transmission barrier is identical. This explanation is highly likely for samples G2 and F14 as Langeveld and colleagues already reported very low amounts of PrP^Sc^ in a Western blot analysis [25]. Additionally, the compatibility of the primary structure, and thus the amino acid sequence of the donor and host PrP^C^, largely influences the susceptibility of a given host. Hence, in particular, during the first passage in a new model with a distant PrP^C^, the transmission barrier might cause constraints and lead to the strain adaption phenomenon [18,31]. However, in this specific case, the transmission barrier was considered low as most isolates carried the same or only slightly deviating PrP^C^ genotypes as the Tgshp IX mouse model. However, it is known that already small variabilities like synonymous polymorphisms (silent mutations) could have a major impact on PrP^Sc^ propagation [32]. The effect of the transmission barrier on the goat scrapie panel has already been shown before in the comprehensive study of Nonno et al., 2020 [20] using transgenic models representing different genotypes (TgVRQ) and species (Tga20) and by the in-depth comparable analysis of TgGoat (Tg501) and Tgbov (Tg110) mice [22]. These factors together with other, yet unknown, components, may also explain the low attack rates and prolonged incubation period seen with three isolates (I2, G1 and UKD2). Nevertheless, the combination of both attack rate and incubation period to one single parameter, namely “transmission efficiency”, allowed for a detailed classification, when compared between several rodent models [20], proving that these characteristics are significant parameters for strain classification.

Investigating a large panel of European goat scrapie isolates allowed us to further conduct a geographical analysis of the circulating classical scrapie prion strains. Such an approach has been applied before in the original (sheep) host but failed to find a geographical connection [33]. In our study, the PrP^Sc^ profiles induced in the Tgshp IX mice by all CS isolates were initially grouped by their country of origin, revealing a surprising homology within most countries. Merely, F16 showed a singular PrP^Sc^ profile within the French group. The homology between the countries was even more surprising. Isolates from Spain, the Netherlands, Greece, Cyprus, the UK and France (excluding F16) provide matching PrP^Sc^ profiles. We therefore summarised these isolates as CS-1. Even more interesting and in accordance with the previous studies [20,22,25], all Italian isolates were homologous as a group and clearly distinguishable in Tgshp IX mice and was thus summarised as CS-2. The unique profile of F16 mentioned above allowed for the definition of a third category (CS-3). This analysis not only shows the geographical variation among European isolates, but it also suggests the occurrence of one major classical scrapie strain circulating in large parts of southern and western Europe. Thus, a parameter defining geographical information should always be taken into consideration when investigating a large panel of isolates. For example, the emergence of a different strain in Italy was formerly associated with a vaccine against *Mycoplasma agalactiae* partly produced from the brain material of sheep [34]. Further, why a different prion strain evolved in one French goat still remains to be clarified. Generally, there are two theories explaining prion strain evolution: For one, it is assumed that selective pressure may induce an alteration of the amplification process so that the now newly evolved PrP^Sc^ is able to cross a given transmission barrier, further adapt and eventually emerge. The second theory states that selective pressure results in the selection of the most suitable PrP^Sc^ conformer already present in a given isolate, which is considered a mixture of different conformers (“sub-strains”) [17,18,32]. Besides polymorphisms of the prion protein gene and the route and dose of exposure, the breed seems to have an influence on the outcome of the disease, at least in cattle [35]. Yet, not all selective factors are known so far. In addition, it should not be forgotten that PrP^Sc^ shows not only a high tenacity in the environment but also against commonly used disinfectants and decontamination procedures. Hence, once shed into the environment, PrP^Sc^ can remain biologically active for a long period of time, and the interference with soil and other environmental matrix might act as a selective trigger promoting certain sub-strains to emerge [36,37,38,39]. Taken together, further epidemiological studies are necessary to trace back the different categories of prion strains in goats seen here.

Previous studies reported that PrP^Sc^ originating from lymph nodes shows different phenotypic characteristics in immunohistochemical analysis. For example, in situ PrP^Sc^ is hardly detectable by N-terminal antibodies, most probably due to partial digestion in the lymphoid cells [40]. To our surprise two lymph node isolates of goats infected with classical scrapie revealed the same pattern as the brain isolates from category CS-1, indicating that the N-terminal part of the protein is not necessarily relevant for strain typing purposes in mouse models. However, one lymph node isolate (UKA1) showed an incomplete attack rate (66.67%) and only mild PrP^Sc^ deposition in the diseased mice. This could also be due to a high transmission barrier or initially low PrP^Sc^ titres, but the unique PrP^Sc^ profile presented by that isolate in Tgshp IX indicates the occurrence of another possibly even a lymph node-associated prion strain. As we still do not have enough data to exclude the interference of the lymph node-amplificated PrP^Sc^ on the profile induced in mice (these samples were not examined in previous reports), we refrain from a direct comparison with the other brain isolates examined and excluded this sample from the categorisation applied here. However, it would be of interest to inoculate the corresponding brain isolate into Tgshp IX mice. That way the profile of the propagated PrP^Sc^ would clarify if lymph nodes can indeed harbour additional prion strains or if the same profile would be induced, indicating the occurrence of a fourth category.

Most importantly the PrP^Sc^ profiles obtained from Tgshp IX mice allowed for a clear-cut discrimination of gtBSE and AS from the diverse classical scrapie isolates examined. Both isolates were used as reference prion strains in the study presented here and interestingly revealed the very same PrP^Sc^ profile and cellular reaction pattern as previously reported for Tgshp IX and Tgshp XI mice (both tg-shARQ) [21,23]. In particular, the very characteristic PrP^Sc^ deposition in the cerebellum for both strains as well as the expansive PL deposits and subpial PrP^Sc^ accumulation for BSE gave convincing proof. These results impressively underline the high stability associated with these prion agents, regardless of the species of origin. In addition, the discrimination of CH1641 by PrP^Sc^ profiling was possible but not as clear-cut as for BSE and AS. For the discrimination of CH1641, the cellular pattern had to be included, which showed a characteristic predominant intracellular (ITNR) PrP^Sc^ accumulation, associated with mild fine extracellular deposits. This pattern is not unusual in Tgshp IX mice and has also been described in the natural sheep host as characteristic of CS [41]. However, the widespread pattern involving almost all brain regions investigated in the mouse model seems to be characteristic of CH1641. Category CS-1 showed some similarities with CH1641, as several isolates, including the Spanish and UK isolates as well as F3 and N3, multifocally revealed a similar pattern predominantly in the caudal brain regions. However, the widespread and distinct dominance induced by the CH1641 isolate was unique. Interestingly, the biochemical analysis of the Spanish isolates published by Nonno and colleagues revealed matching results [20]. The Spanish isolates propagated 19 and 21 kDa bands in the Tgshp IX and tg-bov models as well as in bank voles. The authors assumed that these 19 and 21 kDa bands are sub-strains that exist at different extents in prion isolates and determine certain strain features [20]. The partly overlapping immunohistochemical PrP^Sc^ profile of the S2, S3 and CH1641 isolates clearly supports this theory, indicating that CH1641-like sub-strains might circulate to different extents in European goat scrapie isolates. In this regard, it is of further interest that a similar pattern of an ovine field classical scrapie isolate, showing heavy intracellular deposits, surrounded by fine accumulation, was recently described in Tgshp IX mice [23]. This pattern was detected in a German ARQ/ARQ Merino sheep, clearly indicating that the CH1641-like sub-strains might also occur in sheep and are not confined to caprine CS.

Furthermore, the thorough immunohistochemical investigation of all isolates presented here revealed a unique pattern in three of the nine positive mice inoculated with isolate F11. This PrP^Sc^ profile shares some similarities with BSE in particular because of its multifocal and significant plaque and plaque-like formations. The biochemical phenotype reported before [20] remained inconspicuous with this sample. This finding clearly underlines the importance of IHC for prion strain typing as stated before [9,42]. It is noteworthy that this CS case was, in contrast to the majority of the investigated panel, obtained from an animal trial of goats orally infected with goat scrapie [25]. Moreover, this isolate carries a distinct genotype, showing an amino acid exchange at codon 142 (142IM) as well as the much more frequent silent mutation at codon 240 (240PP). As mentioned above, it is widely accepted that conformational alterations of PrP^Sc^, encoded by a polymorphism in the prion protein gene, have a major impact on defining prion strains, thus hosting strain-specific features [6,18,43,44,45]. This might be the reason for the observed strain shift in this particular case, reflected by the two different cellular patterns for isolate F11. However, the presence of competing sub-strains in the isolate, which were differently propagated by the mice in this group, cannot be ruled out. PrP^Sc^ is known to be highly flexible and adaptive, in particular after transmission to a new host. Serial PMCA modelling interspecies transmission already demonstrated phenotypic shifts in the adapted protein [46,47,48,49]. Of interest in this context are the results published by Krejciova and colleagues who modelled a serial transmission of ovine BSE using cattle and ovine BSE as a seed with a VRQ substrate and observed a switch to the biochemical and biological properties of scrapie after five to six rounds of PMCA [50]. To date, two goats naturally infected with BSE are known: one French goat detected during the 2002 active surveillance programme and one UK case from 1990, retrospectively classified as gtBSE [9,51]. Several authors further hypothesised that BSE prions are derived from either atypical or classical scrapie strains [17,52,53]. As pointed out before, cattle, ovine and caprine BSE induced a similar and very distinctive pattern in Tgshp IX, and for the present F11 isolate, it would be of interest if sub-passages in the Tgshp IX model reproduce a similar PrP^Sc^ profile and cellular pattern or if a further shift to a BSE-like profile would occur. Additional inoculation into other transgenic mouse models representing interspecies transmission, i.e., tg-bov and/or humanised mice, would shed more light on the potential zoonotic nature of the present sub-strain.

## 5. Conclusions

Taken together, the classification and categorisation of CS isolates remains a difficult task with a wide scope for interpretation. It is therefore of utmost importance to take, besides attack rate and incubation period, both immunohistochemical and biochemical parameters into consideration when characterising TSE isolates. However, as reported before [23], the lesion profile does not add robust information for discrimination purposes. Furthermore, with regard to the PrP^Sc^ profile, it has to be emphasised that in the study presented here, additional brain areas as well as cellular patterns were included, and both are not part of the standardised procedures [7,19]. Thus, as recently proposed [23], we suggest adapting the strain typing protocol accordingly by including further brain areas. Most crucial are, at least in the Tgshp IX model, but also in wild-type models as reported before [36], the granular and molecular layer of the cerebellum and the corpus callosum, which allow for, in our study, a clear-cut discrimination not only of AS and gtBSE, but also of certain CS isolates. Additionally, certain cellular patterns like an extensive PL deposition and subpial reaction pattern induced by gtBSE or the distinctive and widespread intraneuronal deposition pattern of CH1641 support the categorisation and should therefore also be included. In this way, the Tgshp IX mouse model is a reliable tool for the discrimination of field prion strains, as previously demonstrated by an in-depth characterisation of several prion strains [23]. We therefore propose to include Tgshp IX (tg-shARQ) mice routinely into strain typing approaches as already suggested by Nonno and colleagues [20].

## Figures and Tables

**Table 1:** Isolate information and transmission features of the TgshpIX (tg-shARQ) mouse model (in parts already published by Nonno et al., 2020 [20]).

Isolate Code	Country of Origin	PrP Genotype ^#^	PrP^Sc^ Type	Attack Rate	Mean Incubation Period (dpi)
I2	Italy	240PP	CS	4/12	410 ± 209
I3	Italy	240PP	CS	7/7	464 ± 72
I4	Italy	211QR, 240PS	CS	3/5	477 ± 58
I5	Italy	240PP	CS	4/5	493 ± 66
I7	Italy	240PP	CS	12/12	417 ± 70
I9	Italy	143HR, 240PS	CS	4/5	400 ± 67
I11	Italy	240PS	CS	12/14	380 ± 116
I12	Italy	240PS	CS	14/15	408 ± 91
I15	Italy	154RH, 240PS	AS	6/7 ^	364 ± 32
S2	Spain	240PS	CS	14/14	222 ± 44
S3	Spain	240PP	CS	13/13	210 ± 33
N1	Netherlands	143HR, 240PS	CS	14/15	276 ± 66
N2	Netherlands	143HR, 240PS	CS	8/9	321 ± 76
N3	Netherlands	240PP	CS	11/14	270 ± 14
F2 *	France	240PS	CS	11/11	177 ± 25
F3	France	240PP	CS	13/15	232 ± 68
F6	France	240PS	CS	13/14	235 ± 38
F10	France	240PS	CS	11/11	251 ± 66
F11 *	France	142IM, 240PP	CS	9/10	297 ± 67
F14	France	142IM, 240PS	CS	9/11 *	380 ± 66
F16	France	240PS	CS	5/9	238 ± 78
gtBSE *	France	211RQ, 240PS	caprine BSE	12/14	230 ± 59
G1	Greece	240PP	CS	2/5 ^	493 ± 45
G2	Greece	240PP	CS	8/9	295 ± 5
G3	Greece	143HR, 240PP	CS	6/6	292 ± 54
G4	Greece	240PP	CS	5/7	399 ± 33
C1	Cyprus	240PP	CS	13/14 ^	295 ± 15
C2	Cyprus	240PP	CS	14/14	292 ± 70
C3	Cyprus	240PP	CS	14/15	292 ± 49
UKA1 ^§^	UK	240PS	CS	6/9	281 ± 128
UKA2	UK	240PS	CS	14/14	224 ± 53
UKB1 ^§^	UK	240PS	CS	7/7	418 ± 68
UKB2	UK	127GS, 240PP	CH1641-like	10/12	176 ± 28
UKC1 ^§^	UK	127GS, 240PP	CS	7/9	363 ± 129
UKD2	UK	211RQ, 240PP	CS	3/7 ^	503 ± 30

Isolate code reflecting the country of origin was first introduced by Langeveld et al. (2019) [25] and will be consecutively used in the main text; * isolates obtained after experimental infection; ^#^ only codons with polymorphisms compared to the homogenous wild-type are given in a one-letter amino acid abbreviation: G = glycine, H = histidine, P = proline, Q = glutamine, R = arginine, S = serine; ^ group containing animals with inconclusive results, and inconclusive results were not included in attack rate (AR) and considered negative for further calculations; ^§^ isolates deriving from lymph nodes, and information on genotype was previously reported [29]; slight deviations in the attack rates and incubation periods to the data previously reported by Nonno et al., 2020 [20] are due to the inclusion of immunohistochemical results, which added a few additional mice to the calculation (the results presented by Nonno et al., 2020 [20] relied solely on the biochemical data of these mice). CS = classical scrapie, AS = atypical scrapie/Nor98; the standard deviation was included with a “±” after the mean incubation period, given in days post infecti.on (dpi).

**Table 2:** List of brain regions used for lesion and PrP^Sc^ profiling.

Short	Brain Region	Fatola et al. (2022) [23]
Grey matter		
NcV	Vestibular nuclei of medulla	G1
Tec	Tectum of cerebellum	G2
CoS	Cortex of the superior colliculus	G3
HTH	Hypothalamus	G4
TH	Thalamus	G5
HC	Hippocampus	G6
Sep	Septal nuclei	G7
CbrA	Cerebral cortex A (at the level of G4 and G5)	G8
CbrB	Cerebral cortex B (at the level of G7)	G9
GrL *	Granular layer of the cerebellar cortex	Cereb/GranLay
MoL *	Molecular layer of the cerebellar cortex	Cereb/MolLay
White matter		
WM	Cerebellar white matter	W1
Dc	White matter in decussation fibres	W2
CpI	Internal capsule	W3
CC	Corpus callosum (at all levels combined)	CC

* examined only in IHC.

**Table 3:** A review of PrP^Sc^ and the transmission features of selected goat TSE isolates in different rodent models including the new results of the study presented here. The animal experiments were conducted within the context of the GoatBSE project. Up to 35 goat field TSE isolates from seven European countries were inoculated in seven well-established rodent models. As reference controls, atypical scrapie, CH1641 and caprine BSE were included.

	Langeveld et al., 2019 [25]	Nonno et al., 2020 [20]	Marín-Moreno et al., 2021 [22]	Ernst et al., 2024
**Bioassay**	None	1st passage in tg-gtARQ (Tg501), tg-shARQ (TgshpIX), tg-shVRQ (Tg338), tg-bov (Tg110), tga20, RIII, Bv109M	1st and 2nd passage in tg-gtARQ (Tg501), tg-bov (Tg110)	1st passage in Tgshp IX (tg-shARQ)
**Methods**	Biochemical characterisation of 38 TSE isolates including discriminatory testing, triplex-WB (mAbs 12B2, Sha31, SAF84), core stability and PK sensitivity Definition of nine molecular PrP^Sc^ parameters for strain categorisation two glycoprofiling approaches three parameters for PK sensitiv ity of N-terminus molecular mass of non-glycosylated PrP^Sc^ double/single profile of triplet banding core stability absence/presence of C-terminal 154–234 PrP sequence (AS discrimination)	Comparative analysis of amplificated PrP^Sc^ induced by 20 isolates Tg-gtARQ/tg-bov: PrP^Sc^ detection via WB using TeSeE ELISA (Bio-Rad) reagents and mAb Sha31 and 12B2 Tg-shARQ: PTA precipitation and subsequent WB detection of PrP^Sc^; mAb L42, P4 Tg-shVRQ/Tga20: TeSeE WB kit (Bio-Rad); mAb Sha31 RIII: triplex-WB procedure; mAb 12B2, Sha31, SAF84 Bv109M: ISS-WB; mAb SAF84 and 12B2	Comparative analysis of amplificated PrP^Sc^ induced by 16 isolates PrP^Sc^ detection via WB using TeSeE ELISA (Bio-Rad) reagents and mAb Sha31 Lesion (H&E) and PrP^Sc^ profiling (pAb R486) of standardised neuroanatomic brain regions (Fraser et al.)	Comparative analysis of amplificated PrP^Sc^ induced by 35 isolates Lesion and PrP^Sc^ profiling of standardised neuroanatomic brain regions [19] Lesion (H&E) and PrP^Sc^ profile (mAb R145) of standardised neuroanatomic brain regions
**Overall Results**	Discrimination of AS, caprine BSE, CH1641 and CS, in total two categories of CS	Discrimination of AS, caprine BSE, CH1641 and CS, in total four categories of CS (C1–4), whereas C3 and C4 show parallels to CS-2, assuming they are subgroups Complete susceptibility in models overexpressing goat and sheep PrP^C^ (tg-shARQ, tg-gtARQ, tg-spVRQ) due to low transmission barrier Low impact of PrP^Sc^ on transmission barrier; IP was either short (~200 dpi) or long (>500 dpi) and highly variable between isolates and rodent models	Discrimination of AS, caprine BSE and CS, in total four categories of CS No transmission of G2 to tg-gtARQ and very low AR in tg-bov in first passage G3 was not transmissible to tg-bov Detection of two prion strains in N3 after 2nd passage in tg-bov UKB2 of Cat. III is CH1641	Discrimination of AS, caprine BSE, CH1641 and CS, in total three groups of CS Mostly high transmission rates with divergent IPs Detection of BSE-like PrP^Sc^ profile in 3/9 mice of one isolate CH1641-like pattern of at least three isolates
**AS (I15)**	low ratio in CEA-ELISA major 8 kDa band in ISS-WB with P4 no binding of Ab SAF84 extremely low PK sensitivity absence of SAF84/Sha31 ratio at 22–24 kDa and C terminaL ~ 154–234 PrP	Characteristic biochemical 8 kDa unglycosylated band remained after 1st passage in tg-gt-ARQ, tg-bov, tg-shARQ, tg-shVRQ, tga20	Not performed	Unique PrP^Sc^ profile and deposition pattern with multifocal coarse accumulation exclusively in molecular layer of cerebellar cortex and CC
**CS Categories**	Category CS-1: Isolates C1-3, F2-3, F6, F10-11, F14, G2-3, G11, G13-16, N1-3, S2-3, UKA2, UKC2 glycoprofile with high monoglycosylated/diglycosylated ratio with mAb Sha31 21K banding low core stability after Gdn-HCl treatment high PK sensitivity of N-terminal PrP^Sc^ P4-epitope (relative to SAF84) Category CS-2: Isolates I2-5, I7, I9, I11-12 intermediate PK sensitivity that ranges between isolates mentioned above and CH1641/BSE isolates	Category 1: Isolates I2-3, I12, F16 distinct TE profile: low TE in tg-gtARQ, tg-shARQ, tg-shVRQ, tg-bov, RIII but unusually high TE in Bv109M no propagation of 19 kDa Category 2: Isolates F3, F6, F10, N3 high TE in small rodent models; intermediate proportions of 19 kDa Category 3: Isolates S2, S3, UKA2 comparable Cat. 2 but also high TE in tg-bov; highest proportions of 19 kDa, considered as subgroup of Cat 2 Category 4: Isolates C1-2, G3, N1 comparable Cat. 2 but low in Tg338; lowest proportions of 19K, considered as subgroup of Cat 2	Category I: Isolates I2, I3, I9, F16 IP slow in tg-gtARQ/fast in tg-bov Non-glycosylated 21 kDa band tg-gtARQ: vacuolisation esp. in BS, MB; glia-associated PrP^Sc^, FINE-COARSE, ITNR tg-bov: lesions esp. in MB, TH, lesser in BS; mostly glia-associated PrP^Sc^ Category II: Isolates F2-3, F6, N3-fast, S2-3 Fast IP 19 kDa in tg-bov, 21 kDa in tg-gtARQ vacuolisation in tg-bov > tg-shARQ esp. in BS, MB PrP^Sc^ deposition ITNR, FINE; COARSE in tg-gtARQ, no PL or glia association Category III: Isolate UKB2 -> CH1641 Category IV: C1-2, G2-3, N1, N3-slow slow IP and 21 kDa tg-gtARQ: moderate vacuolisation in MB, BS; moderate PrP^Sc^ ITNR, severe FINE-COARSE, moderate PL tg-bov: PrP^Sc^ and lesion profile resembles II but lesser vacuolation in BS/more in STRI	Category CS-1: Isolates S2, S3, N1, N2, N3, F2, F3, F6, F10, F11, F14, G1, G2, G3, G4, C1, C2, C3, UKA2, UKB1, UKC1, UKD2 represents EU mean short to intermediate IP with mostly high to complete attack rates PrP^Sc^ mostly in NcV, TH, HTH, CC; to lesser extent in Sep, MB, WM and no accumulation in GrL, MoL, Cbr Category CS-2: Isolates I2, I3, I4, I5, I7, I9, I11, I12 example for geographical variation long IP with varying AR characteristic PrP^Sc^ deposition in GrL and only mild deposits in CC PL in BS Category CS-3: Isolate F16 no PrP^Sc^ in CB and only sporadically in CC; most accumulation in Sep and CbrB Indication for existence of sub-strains: CH1641-like pattern in isolates S2, S3, F11, UKA1 and to lesser extent in N3, F3, UKD2
**CH1641 (UKB2)**	Characteristics: BSE-like banding with low N-terminal ratio in triplex-WB, ISS-WB BSE-like molecular mass of N-band PrP^Sc^ (19 kDa band in triplex-WB) unique banding with SAF84 showing double banding triplet (#1 between 18 and 29 kDa; #2 between 10 and 24 kDa)	Characteristics: Combination of tg-shARQ/tg-gtARQ + tg-shVRQ + tg-bov TE for discrimination of CH1641-like isolates: highest TE tg-shVRQ TE profile resembles Cat. 3 Double unglycosylated band in tg-shVRQ Additional information 19 kDa band of field isolate resulted in 21 kDa in RIII mice indicating hidden 21 kDa sub-strain 19 kDa bands in different rodent models associated with neurotropism in BS LN isolate propagated CS in wt mouse model and is not transmissible to Bv109M	Characteristics: Fast IP and 19 kDa band Double banding of 19 kDa in tg-gtARQ but not in tg-bov tg-gtARQ: vacuolisation in MB and BS, no lesions in Cbr or CS; severe ITNR, intermediate FINE, no PL tg-bov: lesion and PrP^Sc^ profile is comparable to CS Cat. II	Characteristics: Unique ITNR reaction pattern in tg-shARQ with most PrP^Sc^ in WM, BS, CC PL confined to CC No SBPL Sep, Cbr and GrL, MoL remained negative
**Caprine BSE**	Characteristics: distinct glycoprofile with low M/D ratios and smaller N-fraction than CS (Sha31) low PK sensibility indicated by low SAF34/Bar224 ratio characteristic 19K N-band in ISS-WB (SAF84) high core stability after Gdn-HCl treatment	Characteristics: 100% AR in all models Lower TE in tg-shVRQ and Bv109M Uniform propagation of 19K in all models	Characteristics: 100% AR in both models IP shortened in 2nd passage in tg-gtARQ, but remained stable in tg-bov PrP^Sc^ and lesion profile not carried out	Characteristics: Clearly distinguishable PrP^Sc^ profile with prominent, widespread and significant PL, severe SBPL and multifocal accumulation in MolL and GrL of cerebellum
**Geographical Analysis**	CS-2 (IT isolates) as example of geographic variation: possible source in vaccination against *Mycoplasma agalactiae* CS-1 might have existed before but was displaced by probably more dominant CS-2 strain	IT, CY and SP showed homology among isolates from same country but distant profiles when compared to other countries Heterology of isolates from France/Netherlands	Not performed	Most EU isolates revealed parallel features indicating CS-1 as major strain in EU goat populations (CS-2) Italian isolates are clearly distinct from all other isolates and show geographical variation For CS-3 (F16), no geographical variety is known
**Conclusion**	BSE and AS are clearly distinguishable by all methods and in all rodent models from CS CH1641 was distinguishable but combination of at least two methods was necessary; CH1641-like sub-strains could be mixed in different propagations into CS strains CS isolates showed different strain properties and could be divided into at least two to four categories with subcategories depending on methods used			

IT = Italy; NE = Netherlands; FR = France; SP = Spain; GR = Greece; CY = Cyprus; AS = atypical scrapie; CS = classical scrapie; PK = proteinase K; AR = attack rate; ST = mean survival time; mAb = monoclonal antibody; Ab = antibody; IHC = immunohistochemistry; M/D = ratio of monoglycosylated and diglycosylated bands detected in WB; ISS-WB: Western blot (WB) method performed at Istituto Superiore di Sanità (ISS), Department of Food Safety, Nutrition and Veterinary Public Health, Rome, Italy; WBVR-pH8 method = method performed at Wageningen BioVeterinary Research, Lelystad, the Netherlands for investigation of core stability at altered pH levels; NcV = vestibular nuclei of medulla; Tec = tectum of cerebellum; CoS = cortex of superior the colliculus; HTH = hypothalamus; TH = thalamus; HC = hippocampus; Sep = septal nuclei; CbrA = cerebral cortex (at the level of the thalamus and hypothalmus); CbrB = cerebral cortex (at the level of the septal nuclei); WM = cerebellar white matter; Dc = white matter in decussation fibres; DpI = internal capsule; CC = corpus callosum; GrL = granular layer of the cerebellar cortex; MoL = molecular layer of the cerebellar cortex; BS = brain stem; CB = cerebellum; MB = midbrain; DE = diencephalon; Cbr = cerebrum; FB = forebrain; CC = corpus callosum; ITNR/ITGL = intraneuronal, intraglial; FINE = fine particulate; COAL = coalescing; PVASC = perivascular; SBEP = subependymal; PL = plaque and plaque-like formations.

**Table 4:** Overview of parameters most important for discriminating prion strains in Tgshp IX (tg-shARQ) mice.

Isolate	PrP^Sc^ Profile *	Cerebellum MoL/GrL	CC	PL (Other Than CC)	SBPL	ITNR
AS	Negative in standard areas	++/--	(+)	--	--	--
gtBSE	NcV, Tec, HTH, TH, HC, Sep, CbrA+B, WM, DC	++/++	+++	+++	+++	+
CH1641	NcV, CoS, HTH, TH, HC WM, DC	--/--	++	--	--	+++
CS-1 (EU mean)	NcV, CoS, HTH, TH, Sep, WM, DC	--/--	+	--	--	+
CS-2 (Italy)	NcV, CoS, HTH, TH, HC, Sep, CbrB, WM, DV	--/(+)	(+)	+ (mainly in BS)	--	+
CS-3 (F16)	HTH, TH, Sep, CbrB, DC	--/--	--	--	--	+

* peaks of the PrP^Sc^ profile are listed; NcV = vestibular nuclei of medulla; Tec = tectum of cerebellum; CoS = cortex of the superior colliculus; HTH = hypothalamus; TH = thalamus; HC = hippocampus; Sep = septal nuclei; CbrA = cerebral cortex (at the level of thalamus and hypothalamus); CbrB = cerebral cortex (at the level of the septal nuclei); WM = cerebellar white matter; Dc = white matter in decussation fibres; CC = corpus callosum; GrL = granular layer of the cerebellar cortex; MoL = molecular layer of the cerebellar cortex; BS = brain stem; ITNR/ITGL = intraneuronal, intraglial; SBEP = subependymal; PL = plaque and plaque-like formations; -- = negative; + = mild; ++ = moderate; +++ = severe PrP^Sc^ deposition.

## Data Availability

Directly after the publication of this manuscript, the original (raw) data will be publicly available on zenodo.org.

## Supplementary Materials

The following supporting information can be downloaded at: https://www.mdpi.com/article/10.3390/pathogens13080629/s1, Table S1: Categorisation of classical scrapie isolates from goats along attack rates and mean incubation period; Figure S1: Investigated brain areas for lesion and PrP^Sc^ profiling; Figure S2: Correlation between incubation period, attack rate and the scrapie strain categories as well as the reference controls; Figure S3: Characteristic histopathological alteration and immunohistochemical reaction pattern of a TSE infection in the tg-shARQ (Tgshp IX) mouse model; Figure S4: Geographical analysis of caprine classical scrapie isolates per country; Figure S5: Cellular reaction pattern and plaque/plaque-like formations of all investigated brain isolates.
