# Supplementary material for: Characterisation of European Field Goat Prion Isolates in Ovine PrP Overexpressing Transgenic Mice (Tgshp IX) Reveals Distinct Prion Strains
Source: Pathogens. 2024 Jul 27;13(8):629. doi: 10.3390/pathogens13080629 (PMC11357236; doi:10.3390/pathogens13080629)
Supplement: Supplementary file 1 [file pathogens-13-00629-s001.zip › pathogens-3113200-supplementary.pdf]

Table S1. Categorization of classical scrapie isolates from goats along attack rate and mean incubation period.

Category	Isolate code	Attack rate (AR)	AR in %	mean incubation period (dpi)	mean AR
A „short“	**F2**	11/11	100,00%	177 ± 25	90,03%
	**F3**	13/15	86,67%	208 ± 26	
	**S3**	13/13	100,00%	210 ± 33	
	**S2**	14/14	100,00%	222 ± 44	
	**UKA2**	14/14	100,00%	224 ± 53	
	**F6**	13/14	92,86%	235 ± 38	
	**F16**	5/9	55,56%	238 ± 78	
	**F10**	11/11	100,00%	251 ± 66	
	**N3**	11/14	78,57%	270 ± 14	
	**N1**	14/15	93,33%	276 ± 66	
	**UKA1**	6/9	66,67%	281 ± 128	
	**C3**	14/15	93,33%	292 ± 49	
	**G3**	6/6	100,00%	292 ± 54	
	**C2**	14/14	100,00%	292 ± 70	
	**C1**	13/14*	92,86%	295 ± 15	
	**G2**	8/9	88,89%	295 ± 5	
	**F11**	9/11	81,82%	297 ± 67	
B "intermediate"	**N2**	8/9	88,89%	321 ± 76	82,65%
	**UKC1**	7/9	77,78%	363 ± 129	
	**I11**	14/15	93,33%	380 ± 116	
	**F14**	9/11*	81,82%	380 ± 66	
	**G4**	5/7	71,43%	399 ± 33	
C "long"	**I9**	4/5	80,00%	400 ± 67	72,95%
	**I12**	14/15	93,33%	408 ± 91	
	**I2**	4/12	33,33%	410 ± 209	
	**I7**	12/12	100,00%	417 ± 70	
	**UKB1**	7/7	100,00%	418 ± 68	
	**I3**	7/7	100,00%	464 ± 72	
	**I4**	3/5	60,00%	477 ± 58	
	**G1**	2/5*	40,00%	493 ± 45	
	**I5**	4/5	80,00%	493 ± 66	
	**UKD2**	3/7*	42,86%	503 ± 30	

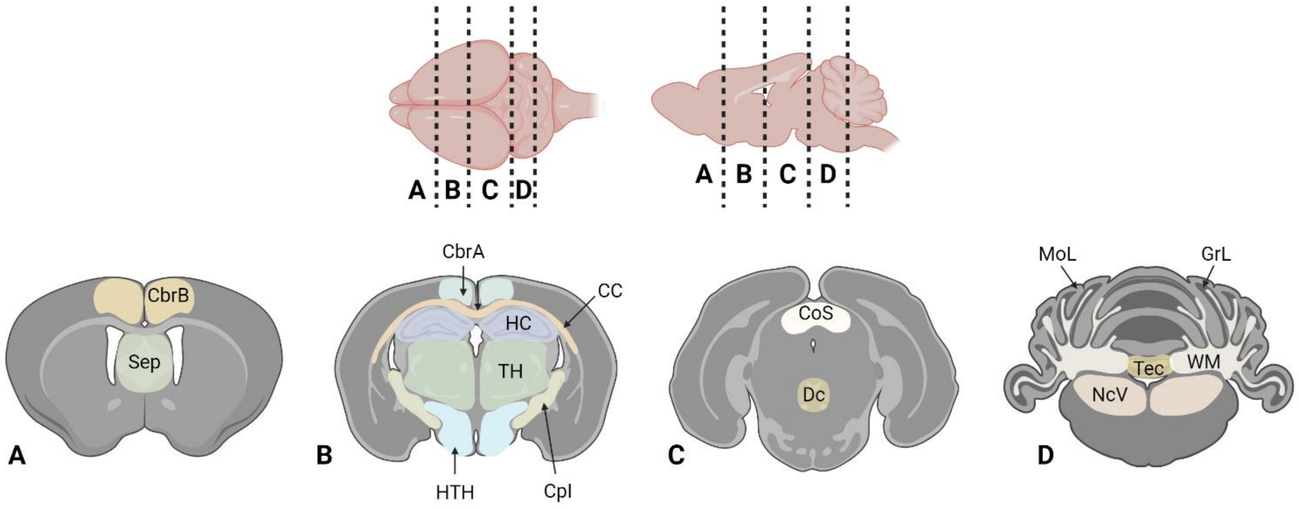

Figure S1. Investigated brain areas for lesion and PrP^{Sc} profiling. Nine grey matter and four white matter areas at four different neuroanatomic levels were analysed for the lesion profile. The PrP^{Sc} profile includes two further areas, the molecular and granular layer of the cerebellar cortex. NcV = vestibular nuclei of medulla; Tec = tectum of cerebellum; CoS = Cortex of superior colliculus; HTH = hypothalamus; TH = thalamus; HC = hippocampus; Sep = septal nuclei; CbrA = cerebral cortex (at the level of thalamus and hypothalmus); CbrB = cerebral cortex (at the level of the septal nuclei); WM = cerebellar white matter; Dc = white matter in decussation fibers; DpI = internal capsule; CC = corpus callosum; GrL = granular layer of the cerebellar cortex; MoL = molecular layer of the cerebellar cortex. Created with BioRender.com.

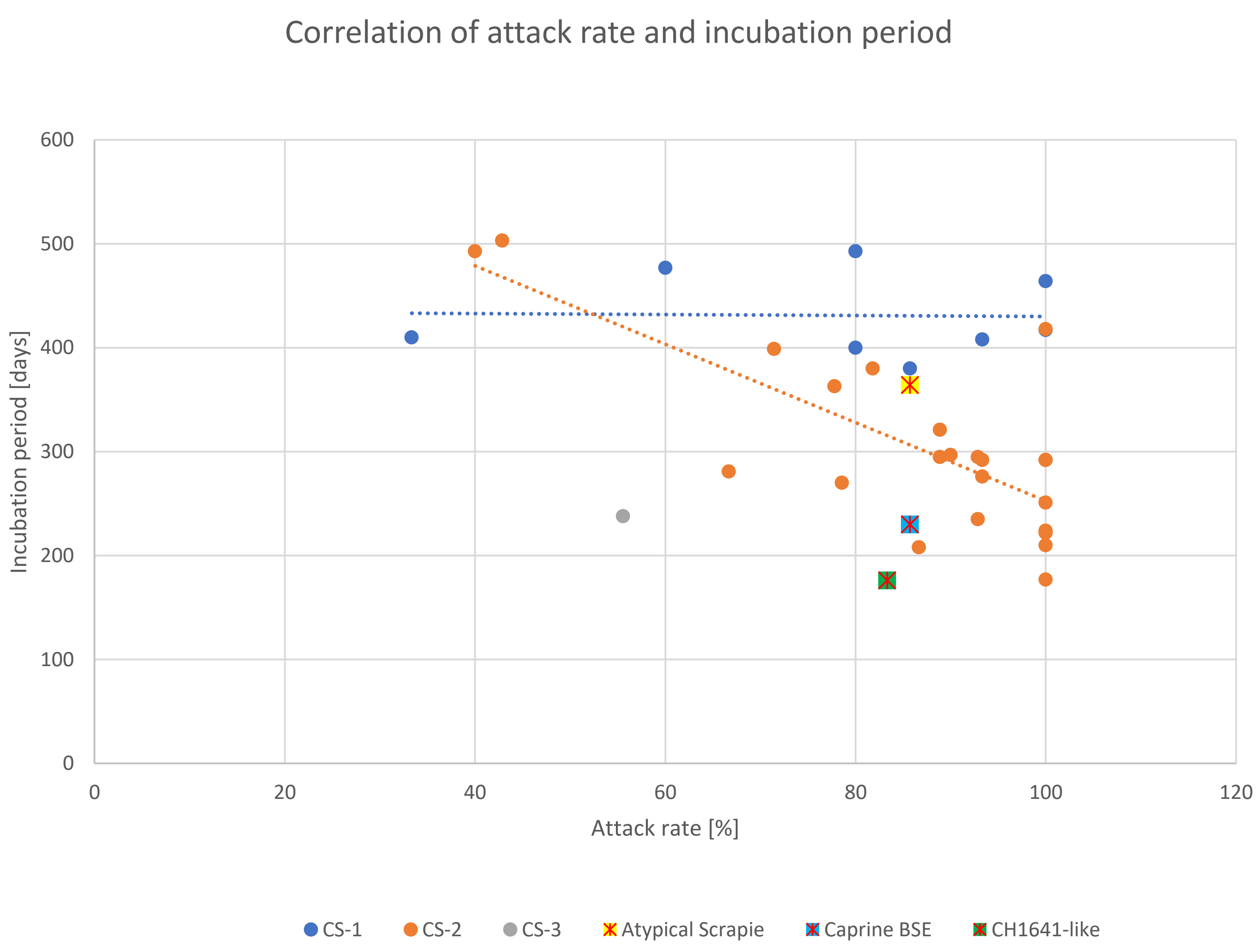

Figure S2: Correlation between incubation period, attack rate and the scrapie strain categories as well as the reference controls. Scrapie isolate F16 (CS-3) is clearly distinct from the other categories. In addition, most of the Italian isolates (CS-2), but I2 are closely clustered.

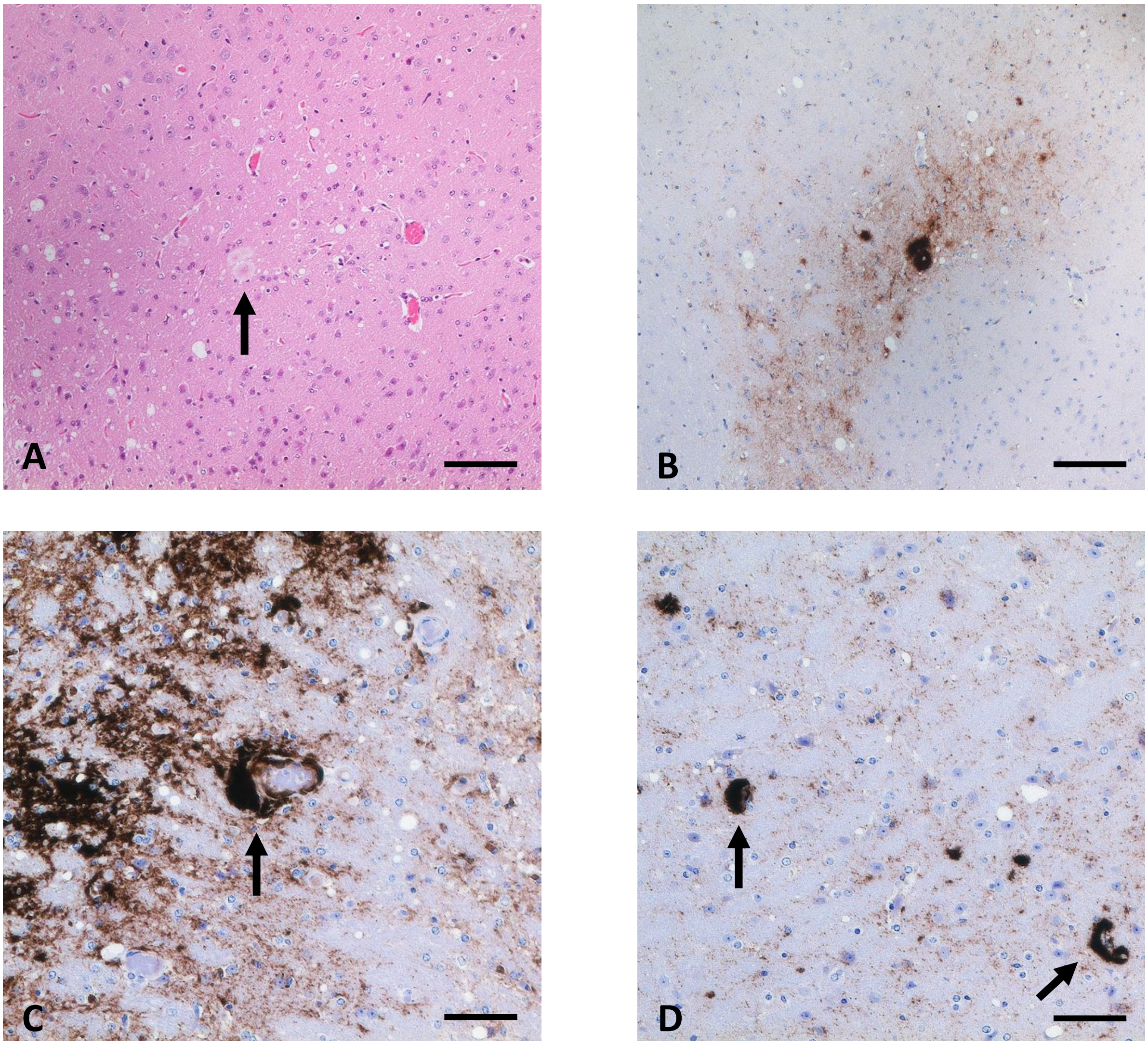

Figure S3: Characteristic histopathological alteration and immunohistochemical reaction pattern of a TSE infection in the tg-shARQ (Tgshp IX) mouse model. **(A)** moderate spongiform encephalopy including plaque formation (arrow), HE-staining, mid brain; **(B)** the same location as shown in A, note that distinct morphological alterations are always associated with distinct PrP^{Sc} accumulation; **(C)** Extracellular coarse granular and coalescing PrP^{Sc} accumulation in the neuropil as well as distinct perivascular PrP^{Sc} deposition (arrow); **(D)** multifocal perivacuolar PrP^{Sc} accumulation (arrows); B-D immunohistochemistry mab R145, Bar 50 µm

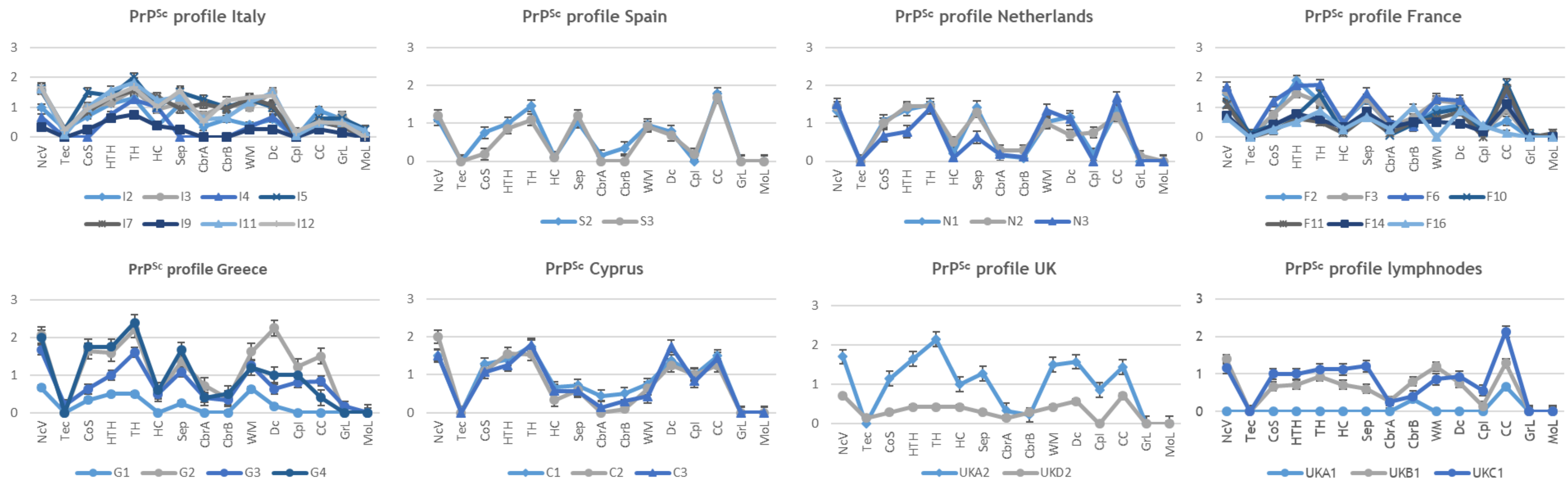

Figure S4. Geographical analysis of caprine classical scrapie isolates per country. Comparative analysis of all classical Scrapie (CS) isolates inoculated into Tgshp IX (tg-shARQ) per country. All CS isolates can be discriminated from reference strains and showed homology to the isolates of the same country, differences seen are merely due to total amounts of PrP^{Sc}. Note, that only Italian isolates show a peak at the GrL, clearly differing from the rest of the isolates. The PrP^{Sc} profile of isolate F16 (France) revealed unique features with almost no PrP^{Sc} accumulation at the CC and WM. NcV = vestibular nuclei of medulla; Tec = tectum of cerebellum; CoS = Cortex of superior colliculus; HTH = hypothalamus; TH = thalamus; HC = hippocampus; Sep = septal nuclei; CbrA = cerebral cortex (at the level of thalamus and hypothalmus); CbrB = cerebral cortex (at the level of the septal nuclei); WM = cerebellar white matter; Dc = white matter in decussation fibers; DpI = internal capsule; CC = corpus callosum; GrL = granular layer of the cerebellar cortex; MoL = molecular layer of the cerebellar cortex. Error bars indicate standard error of means.

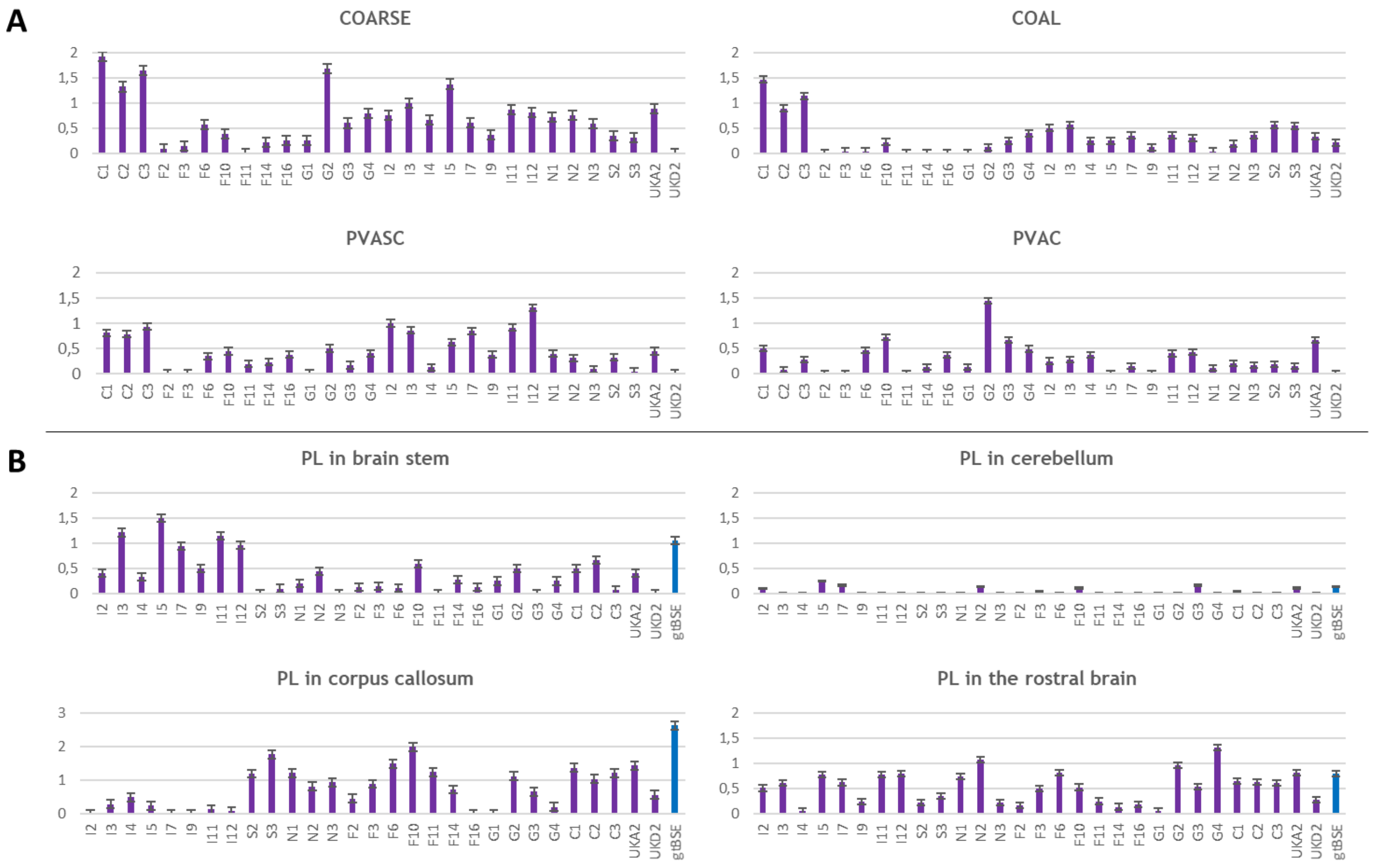

Figure S5. Cellular reaction pattern and plaque/ plaque-like formations of all investigated brain isolates. **(A)** The analysis of coarse (COARSE), coalescing (COAL), perivascular (PVASC) and perivacuolar (PVAC) PrP^{Sc} deposits was most indicative. Coarse deposits were most severe in Cyprian isolates as well as G2, but only rarely seen in French isolates. Accordingly, coalescing pattern is prominently seen in the Cyprian isolates but only rarely found in most of the other isolates and never in French isolates. Perivascular deposits are predominantly seen in the Cyprian and most Italian isolates. Perivacuolar PrP^{Sc} were most prominent in isolate G2 and only occasionally seen in all other isolates. **(B)** Plaque and plaque-like formations (PL) were continuously seen in the brain stem (BS), the corpus callosum and the rostral brain of gtBSE and occurred at lesser extends in all CS isolates. The "rostral brain" encompasses midbrain, diencephalon, fore brain and showed mild PL depositions in all isolates investigated. With exception of the Italian samples, the brain stem (BS) was only mildly affected. I3, I5 and I11 were most affected by PL deposits in the brain stem with relatively lesser extends in the rostral brain. In the CC most PL were detectable in both CS and gtBSE, at mostly intermediate to severe extends. Exceptionally, the Italian isolates, F16, G1 and G4 showed no or only mild PL, in this area. Only mild PL formations were seen occasionally at the level of the cerebellum.
